# Serum hsa_tsr016141 as a Kind of tRNA-Derived Fragments Is a Novel Biomarker in Gastric Cancer

**DOI:** 10.3389/fonc.2021.679366

**Published:** 2021-05-13

**Authors:** Xinliang Gu, Shuo Ma, Bo Liang, Shaoqing Ju

**Affiliations:** ^1^ Department of Laboratory Medicine, Affiliated Hospital of Nantong University, Nantong, China; ^2^ Research Center of Clinical Medicine, Affiliated Hospital of Nantong University, Nantong, China; ^3^ Medical School of Nantong University, Nantong University, Nantong, China; ^4^ Department of Medical Ultrasonics, Affiliated Hospital of Nantong University, Nantong, China

**Keywords:** tRNA-derived small RNAs, gastric cancer, hsa_tsr016141, biomarker, diagnosis

## Abstract

**Background:**

Gastric cancer (GC) is one of the most common malignant tumors globally and the third leading cause of cancer-related death. Currently, the sensitivity and specificity of diagnostic markers for GC are low, so it is urgent to find new biomarkers with higher sensitivity and specificity. tRNA-derived small RNAs are a kind of small non-coding RNAs derived from tRNAs. It is abundant in cancer cells and body fluids. Our goal is to find the differentially expressed tRNA-derived small RNAs in GC to explore their potential as a GC biomarker.

**Methods:**

Quantitative real-time PCR was used to detect the expression level of hsa_tsr016141. The molecular characteristics of hsa_tsr016141 were verified by agarose gel electrophoresis, Sanger sequencing, Actinomycin D Assay, and Nuclear and Cytoplasmic RNA Separation Assay. The diagnostic efficiency of hsa_tsr016141 was analyzed through receiver operating characteristic.

**Results:**

The expression level of hsa_tsr016141 in GC tissues and serum was significantly increased. The serum expression level showed a gradient change between GC patients, gastritis patients, and healthy donors and was positively correlated with the degree of lymph node metastasis and tumor grade. ROC analysis showed that the serum expression level of hsa_tsr016141 could significantly distinguish GC patients from healthy donors or gastritis patients. Besides, the expression level of hsa_tsr016141 in GC patients decreased significantly after the operation (P<0.0001).

**Conclusions:**

Serum hsa_tsr016141 has good stability and specificity and can be used for dynamic monitoring of GC patients, suggesting that serum hsa_tsr016141 can be a novel biomarker for GC diagnosis and postoperative monitoring.

## Introduction

Gastric cancer (GC) is one of the most common malignancies globally, and more than 1 million people are newly diagnosed with GC worldwide every year (1). GC can occur in any part of the stomach. The vast majority of GC is adenocarcinoma, originating from the most superficial glands or mucous membrane of the stomach (2). Although the morbidity and mortality of GC have declined worldwide in the past 50 years, it is still the third leading cause of cancer-related death (2, 3). The early symptoms of GC are not obvious, and the symptoms of benign gastric diseases such as gastritis and gastric ulcers are easy to be ignored. Due to the lack of specific early diagnostic markers, patients often miss the best opportunity for treatment (4). Therefore, there is an urgent need to find accurate biomarkers and therapeutic targets. Some studies have shown that non-coding RNAs can be used as a biomarker of tumor diagnosis and prognosis. For example, the expression level of miR-425-5p was upregulated in cervical cancer and could be used as a promising prognostic biomarker for cervical cancer (5). In addition, HIF1A-AS2 was a dependable predictor of malignancy and prognosis in GC, and circEHBP1 could act as a promising biomarker for lymphatic metastasis in bladder cancer (6, 7). However, the sensitivity and specificity of the existing diagnostic markers for GC are low, so it is of high clinical significance to search for new biomarkers with high sensitivity and specificity. In recent years, novel small non-coding RNAs called tRNA-derived small RNAs (tsRNAs) have gradually attracted attention, and we have a strong interest in whether they can be used as a promising biomarker.

The discovery of tsRNAs can be traced back to the late 1970s. It was initially considered to be the product of random degradation of tRNAs and did not attract widespread attention (8, 9). However, with the rapid development of high-throughput sequencing technology and the exploration of the role of small RNAs in gene regulation, the research related to small RNAs is increasing (10, 11). In recent years, a large number of experiments and studies have proved that tsRNAs are a derivative fragment produced by specific cleavage of pre-tRNAs or mature tRNAs in a specific environment (12), which can be divided into two types: tRNA-derived fragments (tRFs) and tRNA halves (tiRNAs) according to the cutting position on the pre-tRNAs or mature tRNAs (13, 14). tsRNAs can mainly inhibit the activity of peptidyl transferase by binding to small ribosomal subunits, thus affecting the occurrence and development of tumors. It can also inhibit protein translation through the mechanism of protein sponge (15). Previous studies showed that 5′-tiRNA^Val^ could inhibit the FZD3/Wnt/β-Catenin signaling pathway to suppress the proliferation, migration, invasion, and other functions of tumor cells (16). Zhang found that tRF-3019a could serve as a promising biomarker for GC and target FBXO47 to promote the proliferation, migration, and invasion of GC cells (17). Tong found that the expression of tRF-3017A was increased in GC tissues and cell lines, and it could regulate the migration and invasion of GC cells by targeting NELL2 (18). The research on tsRNAs is mainly on the mechanism presently, but there are few studies on whether they can be good biomarkers in serum. tRFs and tiRNAs have the characteristics of high expression and high stability in a variety of body fluids and are related to a variety of pathological conditions. They have strong discrimination between cancer patients and normal controls, which makes them have the potential to become a new biomarker. Besides, tRFs are usually about 14-30 nucleotides (nt) in length, which was similar to that of microRNAs. According to the different digesting positions of Angiogenin, Dicer, or other RNases on the mature tRNA or pre-tRNA, they can be divided into five types, 5’tRF, 3’tRF, i-tRF, tRF-2 and tRF-1 (15). Among them, 5’tRF has 5′- phosphate, while 3’tRF has 3′-hydroxyl groups, these tRFs can suppress the translation of mRNA by combining the 5’ or 3’ ends with the conservative region of 3’-UTRs in mRNAs (19). They have attracted more and more attention over the past few years (20). Overall, a growing body of evidence suggests that abnormal expression of tRFs is associated with human tumor disease and may become new diagnostic biomarkers (21). Therefore, we focused on 5’tRF and 3’tRF in all kinds of tsRNAs for the exploration of new GC biomarkers.

In this study, we focused on the potential of hsa_tsr016141 as a tumor marker of GC and analyzed its value in clinical application. We found that hsa_tsr016141 can be used as a useful tumor marker. Compared with normal controls, the expression level of hsa_tsr016141 in tissue and serum of patients with GC was upregulated. Its expression level increased with the increase of lymph node metastasis and tumor grade, showing good diagnostic efficacy for GC patients. It has good stability and specificity in clinical application, and the diagnostic efficiency was the highest after combined diagnosis. At the same time, hsa_tsr016141 can effectively track the postoperative condition of GC, play a dynamic monitoring role in patients with GC. Therefore, hsa_tsr016141 provides a potential possibility for early diagnosis and postoperative monitoring of GC.

## Materials and Methods

### Human Serum Samples and Tissue Specimens

All the serum samples in this study included 130 cases of GC patients, 110 cases of healthy donors, 50 cases of gastritis patients, 63 postoperative samples from patients with GC after the operation, and 20 cases of breast cancer (BC), colorectal cancer (CRC), lung cancer (LC), and thyroid cancer (TC) patients were collected in the Clinical Laboratory, Affiliated Hospital of Nantong University. In this study, all patients with GC, BC, CRC, LC, TC and gastritis were clinically diagnosed and did not receive radiotherapy or chemotherapy before. 20 pairs of GC specimens were collected in the Department of Pathology of Affiliated Hospital of Nantong University. All tissue specimens were diagnosed by pathologists as GC and placed immediately into an RNA fixator Bioteke (Nantong, China), after resection and stored at −80°C refrigerator. All participants had obtained informed consent prior to the clinical trial and consented to publication. All of the above samples were collected in accordance with the Code Ethics of the World Medical Association from September 2016 to January 2021. This study was approved by the ethics committee of the local hospital (ethical review report number: 2018-L055).

### Cell Culture

Human GC cell lines (MKN-45, AGS, BGC-823, and HGC-27) and human gastric epithelial cells (GES-1) were purchased from the Chinese Academy of Sciences (Shanghai, China). All of the cells were cultured in RPMI-1640 medium (Corning, USA) with 10% Fetal bovine serum (FBS) (Gibco, USA) and 1% penicillin and streptomycin were added. All cells were culture at 37°C, 5%CO_2_.

### Total RNA Extraction and cDNA Synthesis

Total RNA in serum of GC patients was extracted using Total RNA Pure and Isolation Kit with Spin Column (BioTeke, Beijing, China), and total RNA in tissues and cells was extracted by TRIzol Reagent (Invitrogen, Germany). Then 20µL cDNA was generated by Revert Aid RT Reverse Transcription Kit (Thermo Fisher Scientific) from 10µL total RNA solution, which was incubated at 42°C for 60 min and 70°C for 5 min.

### Real-Time Fluorescent Quantitative PCR

Roche Light Cycler 480 (Roche, Switzerland) was used for the qRT-PCR reaction. The reaction system included 10μL of SYBR Green I Mix (Roche), 1μL of primer, 5μL of cDNA, and 3μL of enzyme-free Water. U6 was used to standardize the relative expression of hsa_tsr016141, and the expression level was calculated through the 2^−ΔΔCT^ method. All primers used in this study were synthesized by RiboBio (Guangzhou, China).

### Actinomycin D Assay

Actinomycin D at a concentration of 1000μg/mL was diluted to 2.5μg/mL by Complete Medium. The complete medium in the six-well plate was replaced with the above-diluted medium containing actinomycin D and cultured 24 hours. TRIzol was added successively at 0, 2, 4, 8, 12, and 24 hours to extract RNA.

### Nuclear and Cytoplasmic RNA Separation Assay

5×10^6^ cells were digested by trypsin and placed in a small centrifuge tube after digestion. Then, according to the procedure of the PARIS™ Kit (Thermo Fisher Scientific), the cells were isolated and extracted into 60 μL Nuclear RNA and Cytoplasmic RNA and stored in the refrigerator at -80°C.

### Statistical Analysis

Statistical analysis was conducted by SPSS Statistics Version 20.0 (IBM SPSS Statistics, Chicago, USA) and GraphPad Prism v8.0 (GraphPad Software, San Diego, CA). The expression level of hsa_tsr016141 in different groups was expressed by mean ± SD. Two-sided Test was used to compare two independent groups, while one-way analysis of variance was used when compared multiple independent groups. ROC curve area under the curve (AUC) was established and calculated to evaluate the diagnostic performance. P<0.05 was considered to have statistical significance.

## Results

### Database and Tissues Screening of hsa_tsr016141

**Figure 1:**
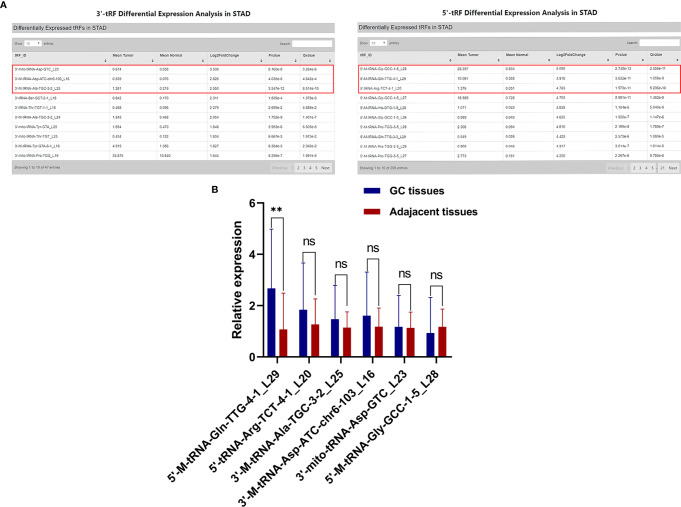
Screening of hsa_tsr016141. **(A)** Three 3’tRF and 5’tRF were sorted by the standard of the most significant log_2_ fold change, and their P values were all less than 0.05.** (B)** Expression levels of 6 tRFs in GC tissues. **P < 0.01, ns P > 0.805.

To explore whether tsRNAs can be used as a good biomarker for GC, we carried out screening in the OncotRF database (http://bioinformatics.zju.edu.cn/OncotRF/). We sorted by the standard of log_2_ fold change and selected the top three 3’tRF and 5’tRF with log_2_ fold change, and their P values were all less than 0.05. The specific information can be found in **Figure 1A**. Subsequently, the qRT-PCR analysis was performed to verify the differences in the expression levels of these six tRFs between 20 GC tissues and their matching adjacent non-tumor tissues. We found that the expression of 5’-M-tRNA-Gln-TTG-4-1_L29 increased significantly in GC. However, the expression level of 3’-M-tRNA-Asp-ATC-chr6-103_L16, 3’-mito-tRNA-Asp-GTC_L23, 3’-M-tRNA-Ala-TGC-3-2_L25, 5’-M-tRNA-Gly-GCC-1-5_L28, and 5’-tRNA-Arg-TCT-4-1_L20 did not differ significantly between GC tissues and their adjacent non-tumor tissues **(** **Figure 1B** **)**. Subsequently, we followed up an in-depth study of it and named it as hsa_tsr016141 according to its naming rules in tsRBase (http://tsrbase.org/).

### hsa_tsr016141 Is a Kind of tRFs

**Figure 2:**
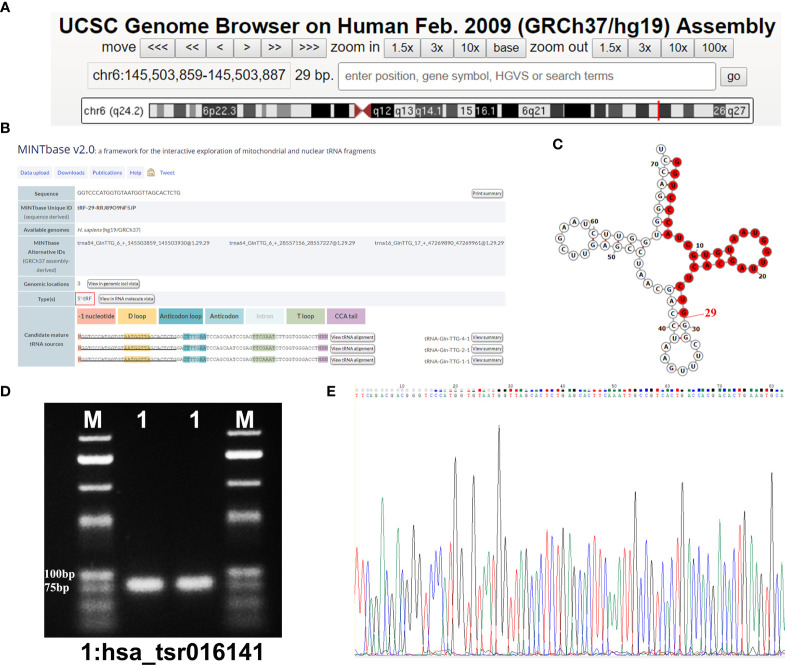
Hsa_tsr016141 is a kind of tRFs. **(A)** hsa_tsr016141 was mapped to chromosome 6q24.2 with coordinates of 145,503,859–145,503,887. **(B)** hsa_tsr016141 was a 5’tRF of tRNA-Gln-TTG, which was processed from tRNA-Gln-TTG-1-1, tRNA-Gln-TTG-2-1, and tRNA-Gln-TTG-4-1. **(C)** Taking tRNA-Gln-TTG-4-1 as an example, the cleavage site of hsa_tsr016141 was located above the anticodon loop. **(D)** The product of qRT-PCR was run on 2% agarose gel, showing a single electrophoresis band. **(E)** The product of qRT-PCR was confirmed by Sanger sequencing that contained the full-length sequence of hsa_tsr016141.

Using the UCSC Genome Browser database, we found that hsa_tsr016141 was mapped to chromosome 6q24.2 with coordinates of 145,503,859–145,503,887 **(** **Figure 2A** **)**. According to the basic information of hsa_tsr016141 in OncotRF Database and MINTbase v2.0 (http://cm.jefferson.edu/MINTbase/.). We determined that it is a tRNA-derived fragment with a length of 29nt (5’-GGTCCCATGGTGTAATGGTTAGCACTCTG-3’). Hsa_tsr016141 was a 5’tRF of tRNA-Gln-TTG, which was processed from tRNA-Gln-TTG-1-1, tRNA-Gln-TTG-2-1 and tRNA-Gln-TTG-4-1 **(** **Figure 2B** **)**. The cleavage sites are all located above the anticodon loop **(** **Figure 2C** **)**. Then, the product of qRT-PCR was detected by agarose gel electrophoresis (AGE), showing a single electrophoresis band of about 80bp **(** **Figure 2D** **)**, and confirmed by sequencing that the product contained the full-length sequence of hsa_tsr016141 **(** **Figure 2E** **)**.

### Characterization of hsa_tsr016141 and Its Advantage as a Biomarker for GC

**Figure 3:**
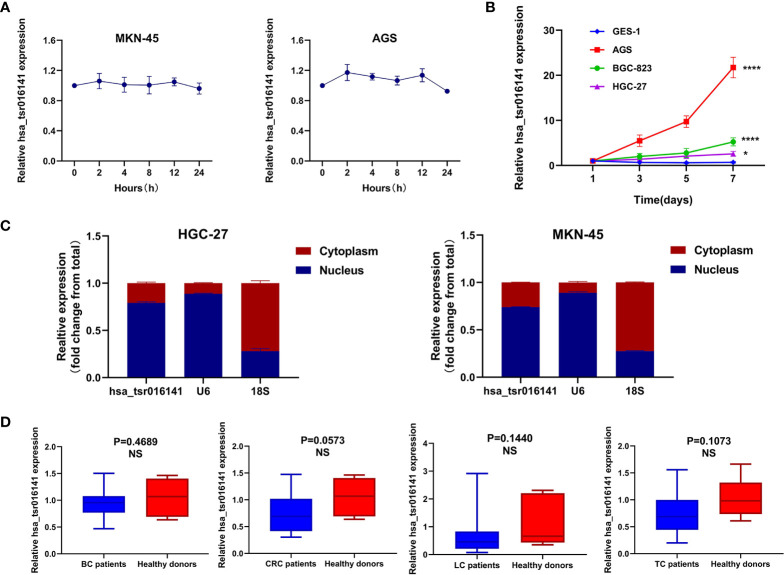
Characterization and advantage of hsa_tsr016141 as a GC biomarker**. (A)** The good stability of hsa_tsr016141 was confirmed by actinomycin D assay. **(B)** The expression level of hsa_tsr016141 in culture supernatants of AGS and BGC-823 increased with time compared to GES-1. **(C)** The location of hsa_tsr016141 in HGC-27 and MKN-45 cells was detected by Nuclear and Cytoplasmic RNA Separation Assay. **(D)** The expression level of hsa_tsr016141 had no significant difference in other tumors. *P < 0.05, ****P < 0.0001, NS P > 0.05.

To investigate the stability of hsa_tsr016141, we conducted an actinomycin D experiment, due to actinomycin D can inhibit RNA production by inhibiting the activity of RNA polymerase. We cultured MKN-45 cells and AGS cells in a medium containing actinomycin D for 24 hours. Through qRT-PCR, we found that the expression level of hsa_tsr016141 was not significantly decreased, which confirmed the good stability of hsa_tsr016141 **(** **Figure 3A** **)**. Subsequently, to investigate the origin of hsa_tsr016141 in serum from GC cells, three GC cells AGS, BGC-823, HGC-27, and gastric epithelial cell GES-1 were cultured for 7 days, and supernatants were collected regularly. The result showed that the expression level of hsa_tsr016141 in AGS, BGC-823, and HGC-27 supernatants increased with the extension of culture time, especially in AGS and BGC-823 cells. However, the expression of hsa_tsr016141 in GES-1 supernatant did not change significantly. It is suggested that hsa_tsr016141 may be released into the blood by GC cells and has the potential to be used as a biomarker **(** **Figure 3B** **)**. Besides, we selected MKN-45 and HGC-27 cells for Nuclear and Cytoplasmic RNA Separation Assay and found that most hsa_tsr016141 in the two kinds of cells was located in the nucleus **(** **Figure 3C** **)**. Then, we collected 20 cases of BC, CRC, LC, TC, and 20 cases of healthy donors serum samples, respectively, to detect the expression level of hsa_tsr016141 in these four other cancers. Through the qRT-PCR, we found that there was no statistically significant difference between serum of these four kinds of cancer patients and healthy donors (P-values were 0.4689, 0.0573, 0.1440, and 0.1073) (**Figure 3D**), but we previously found that the expression level of hsa_tsr016141 was significantly increased in GC. In conclusion, the above evidence proves that hsa_tsr016141 has good characteristics as a biomarker for GC.

### Methodological Evaluation and Biomarker Potential of hsa_tsr016141 Detection in Serum Samples

**Figure 4:**
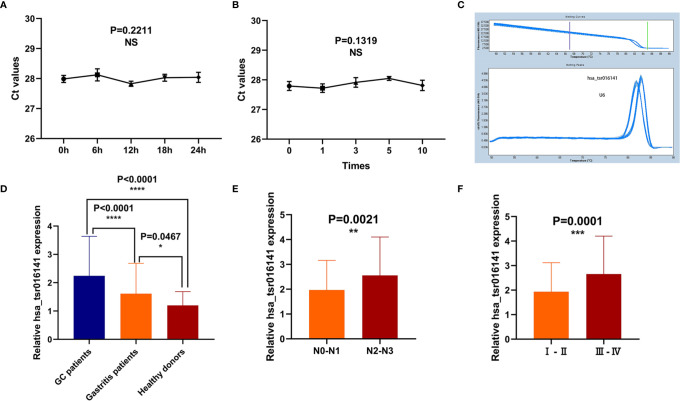
Methodological evaluation and biomarker potential of hsa_tsr016141. **(A, B)** The detection method of hsa_tsr016141 would not be easily affected and had good stability and repeatability. **(C)** The melting curve of hsa_tsr016141. **(D)** The expression level of hsa_tsr016141 in GC patients (n=130), gastritis patients (n=50), and healthy donors (n=110). **(E)** The expression levels of hsa_tsr016141 in serum samples of GC at different stages of lymph node metastasis. **(F)** The expression levels of hsa_tsr016141 in serum samples of GC at different stages of tumor grade. *P < 0.05, **P < 0.01, ***P < 0.001, ****P < 0.0001, NS P > 0.05.

To explore whether the detection of hsa_tsr016141 can be used in clinical analysis, we first made an evaluation of its detection methods comprehensively. We used the mixed serum to determine the detection accuracy of hsa_tsr016141 and found that the intra-assay coefficient of variation (CV) and the inter-assay CV performed well **(** **Table 1** **)**. Subsequently, the mixed serum samples were placed at room temperature for 0, 6, 12, 18, and 24 hours and freeze-thawed repeatedly for 0, 1, 3, 5, and 10 times, and then the relative expression level of hsa_tsr016141 was detected. The results showed no significant difference in its expression level, which revealed that the detection method of hsa_tsr016141 would not be affected by these factors and had good stability and repeatability **(** **Figures 4A, B** **)**. Besides, the smooth and unimodal specific melting curve also shows the accuracy and specificity of this method **(** **Figure 4C** **)**. In conclusion, the detection method of hsa_tsr016141 is suitable for clinical analysis.

**Table 1:** The Intra-Assay CV and the Inter- Assay CV of hsa_tsr016141.

	hsa_tsr016141	U6
**Intra assay CV,%**	**2.14**	**2.95**
**Inter assay CV,%**	**1.94**	**3.13**

CV, coefficient of variation.

In order to explore the diagnostic significance of hsa_tsr016141 in the serum of GC, we collected serum samples from 130 patients with GC, 50 patients with gastritis, and 110 healthy controls to explore the difference in their expression levels. We found that the expression level of hsa_tsr016141 in the serum of patients with GC was significantly higher than that of healthy donors (P<0.0001). In addition, the expression level of hsa_tsr016141 in the serum of patients with gastritis was higher than that of healthy donors (P=0.0467) but significantly lower than that of patients with GC (P<0.0001) **(** **Figure 4D** **)**. In order to further explore whether the expression level of hsa_tsr016141 in the serum is related to tumor metastasis or tumor progression, we classified the expression level of hsa_tsr016141 in serum of patients with GC according to lymph node metastasis and TNM stage. The results showed that the expression level of hsa_tsr016141 increased with the increase of lymph node metastasis. Also, the expression level increased as the tumor grade increased (P=0.0001). These results indicated that the serum expression level of hsa_tsr016141 was elevated in patients with GC and positively correlated with lymph node metastasis and tumor grade **(** **Figures 4E, F** **)**.

### Comparison and Combination of Serum hsa_tsr016141 and Other Tumor Markers in the Diagnosis of GC

**Figure 5:**
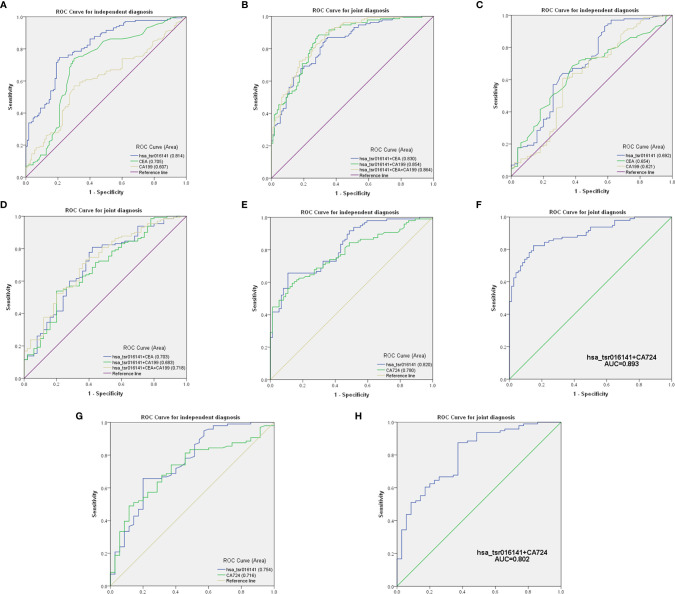
Comparison and combination of serum hsa_tsr016141 and other tumor markers in the diagnosis of GC. **(A)** ROC curve analysis of hsa_tsr016141, CEA, and CA199 in independent diagnosis of GC patients and healthy donors. **(B)** ROC curve analysis of hsa_tsr016141, CEA, and CA199 in joint diagnosis of GC patients and healthy donors. **(C)** ROC curve analysis of hsa_tsr016141, CEA, and CA199 in independent diagnosis of GC patients and gastritis patients. **(D)** ROC curve analysis of hsa_tsr016141, CEA, and CA199 in joint diagnosis of GC patients and gastritis patients. **(E)** ROC curve analysis of hsa_tsr016141 and CA724 in independent diagnosis of GC patients and healthy donors. **(F)** ROC curve analysis of hsa_tsr016141 and CA724 in joint diagnosis of GC patients and healthy donors. **(G)** ROC curve analysis of hsa_tsr016141 and CA724 in independent diagnosis of GC patients and gastritis patients. **(H)** ROC curve analysis of hsa_tsr016141 and CA724 in joint diagnosis of GC patients and gastritis patients.

Firstly, we further discussed the diagnostic characteristics and efficacy of hsa_tsr016141 as a potential biomarker for GC. It is well known that carcinoembryonic antigen (CEA) and Carbohydrate antigen199 (CA199) are commonly used clinical tumor markers. We used 130 GC patients and 110 healthy donors to perform ROC analysis on hsa_tsr016141, CEA, and CA199 to determine the diagnostic efficacy of hsa_tsr016141 in GC serum. The ROC curve showed that the AUC of hsa_tsr016141 was 0.814 (95% confidence interval (CI): 0.760-0.867), which was higher than 0.705 (95% CI: 0.637-0.774) of CEA and 0.607 (95% CI: 0.535-0.678) of CA199 **(** **Figure 5A** **)**. Meanwhile, the sensitivity (75%), specificity (78%), accuracy (76%), positive predictive value (80%), and negative predictive value (72%) of hsa_tsr016141 were also higher than those of CEA and CA199. Subsequently, we analyzed the efficacy of joint diagnosis and found that AUC increased to 0.830 after the combination of hsa_tsr016141 and CEA, and 0.854 after combining of hsa_tsr016141 and CA199. The AUC of the combination of the three was the highest, reaching 0.864 **(** **Figure 5B** **)**. Simultaneously, the sensitivity of joint diagnosis was also increasing, and the sensitivity of the combination of the three is up to 90%, which was higher than that of each tumor marker **(** **Table 2** **)**. The above analysis results indicate that hsa_tsr016141 may have the potential to become a biomarker of GC and, combined with other tumor markers, can improve the diagnostic efficiency of a single tumor marker. Next, we analyzed whether the expression level of hsa_tsr016141 could distinguish patients with GC from those with gastritis. ROC analysis was performed in 130 patients with GC and 50 patients with gastritis. The ROC curve showed that the AUC of hsa_tsr016141 was 0.692 (95% CI: 0.599-0.785), which was higher than 0.654 of CEA (95% CI: 0.567-0.741) and 0.621 of CA199 (95% CI: 0.522-0.720) **(** **Figure 5C** **)**. The AUC of hsa_tsr016141 combined with CEA was 0.703, and that of CA199 was 0.683. The highest AUC was the combination of the three of 0.718, and the sensitivity was 81%, indicating that hsa_tsr016141 can also be used as a biomarker to distinguish GC from gastritis **(** **Figure 5D** and **Table 3** **)**.

**Table 2:** Use the expression levels of hsa_tsr016141, CEA, and CA199 to distinguish GC patients from healthy donors.

	SEN,%	SPE,%	ACCU,%	PPV,%	NPV,%
hsa_tsr016141	0.75(97/130)	0.78(86/110)	0.76(183/240)	0.80(97/121)	0.72(86/119)
CEA	0.68(89/130)	0.73(80/110)	0.70(169/240)	0.75(89/119)	0.66(80/121)
CA199	0.60(78/130)	0.64(70/110)	0.62(148/240)	0.66(78/118)	0.57(70/122)
hsa_tsr016141+CEA	0.86(112/130)	0.66(73/110)	0.77(185/240)	0.75(112/149)	0.80(73/91)
hsa_tsr016141+CEA+CA199	0.90(117/130)	0.66(73/110)	0.79(190/240)	0.76(117/154)	0.66(73/111)

SEN, sensitivity; SPE, specificity; ACCU, overall accuracy; PPV, positive predictive value; NPV, negative predictive value.

**Table 3:** Use the expression levels of hsa_tsr016141, CEA, and CA199 to distinguish GC patients from gastritis patients.

	SEN,%	SPE,%	ACCU,%	PPV,%	NPV,%
hsa_tsr016141	0.64(83/130)	0.68(34/50)	0.65(117/180)	0.84(83/99)	0.52(34/65)
CEA	0.68(89/130)	0.62(31/50)	0.67(120/180)	0.82(89/108)	0.43(31/72)
CA199	0.60(78/130)	0.68(34/50)	0.62(112/180)	0.83(78/94)	0.40(34/86)
hsa_tsr016141+CEA	0.77(100/130)	0.60(30/50)	0.68(130/180)	0.83(100/120)	0.50(30/60)
hsa_tsr016141+CEA+CA199	0.81(105/130)	0.52(26/50)	0.69(131/180)	0.81(105/129)	0.51(26/51)

SEN, sensitivity; SPE, specificity; ACCU, overall accuracy; PPV, positive predictive value; NPV, negative predictive value.

Secondly, in patients with GC at different stages, the levels of serum CEA, CA199, and Carbohydrate antigen724 (CA724) may be increased (22), but the positive rate of CA724 is generally higher than that of CEA and CA199 (22–24). Therefore, we further studied and analyzed the difference in diagnostic efficacy between hsa_tsr016141 and CA724. CA724 was detected in 96 of 130 GC patients and 74 of 110 healthy donors. Therefore, we performed a ROC analysis on these 96 GC patients and 74 healthy donors. The results showed that the AUC of hsa_tsr016141 was 0.820 (95% CI: 0.759-0.881), which was slightly higher than 0.780 of CA724 (95% CI: 0.712-0.848) **(** **Figure 5E** **)**. The sensitivity (66%) and specificity (89%) of hsa_tsr016141 were also slightly higher than that of CA724. Subsequently, we combined the two and found that the AUC increased significantly, reaching 0.893 **(** **Figure 5F** **)**, and the sensitivity was also higher than the single index reached 82%, indicating that both hsa_tsr016141 and CA724 have high diagnostic efficiency, and after the combination of the two, the diagnostic efficiency would be significantly improved **(** **Table 4** **)**. CA724 was detected in 35 of 50 gastritis patients, and then we performed a ROC analysis on these 96 GC patients and 35 gastritis patients. It was found that the AUC of hsa_tsr016141 was 0.754 (95% CI: 0.656-0.851), which was slightly higher than 0.716 of CA724 (95% CI: 0.622-0.810) **(** **Figure 5G** **)**. The AUC increased to 0.802, and the sensitivity significantly increased to 88% after the combination of the two **(** **Figure 5H** **)**. In conclusion, hsa_tsr016141 has higher diagnostic efficiency than CA724, and the combination of the two would further improve the diagnostic efficiency **(** **Table 5** **)**.

**Table 4:** Use the expression levels of hsa_tsr016141 and CA724 to distinguish GC patients from healthy donors.

	SEN,%	SPE,%	ACCU,%	PPV,%	NPV,%
hsa_tsr016141	0.66(63/96)	0.89(66/74)	0.76(129/170)	0.89(63/71)	0.67(66/99)
CA724	0.64(61/96)	0.77(57/74)	0.69(118/170)	0.78(61/78)	0.57(57/92)
hsa_tsr016141+CA724	0.82(79/96)	0.85(63/74)	0.84(142/170)	0.88(79/90)	0.79(63/80)

SEN, sensitivity; SPE, specificity; ACCU, overall accuracy; PPV, positive predictive value; NPV, negative predictive value.

**Table 5:** Use the expression levels of hsa_tsr016141 and CA724 to distinguish GC patients from gastritis patients.

	SEN,%	SPE,%	ACCU,%	PPV,%	NPV,%
hsa_tsr016141	0.66(63/96)	0.80(28/35)	0.69(91/131)	0.90(63/70)	0.46(28/61)
CA724	0.64(61/96)	0.69(24/35)	0.65(85/131)	0.85(61/72)	0.41(24/59)
hsa_tsr016141+CA724	0.88(84/96)	0.63(22/35)	0.81(106/131)	0.87(84/97)	0.65(22/34)

SEN, sensitivity; SPE, specificity; ACCU, overall accuracy; PPV, positive predictive value; NPV, negative predictive value.

### Correlation Between Serum hsa_tsr016141 Expression and Clinicopathological Parameters in Patients With GC

**Figure 6:**
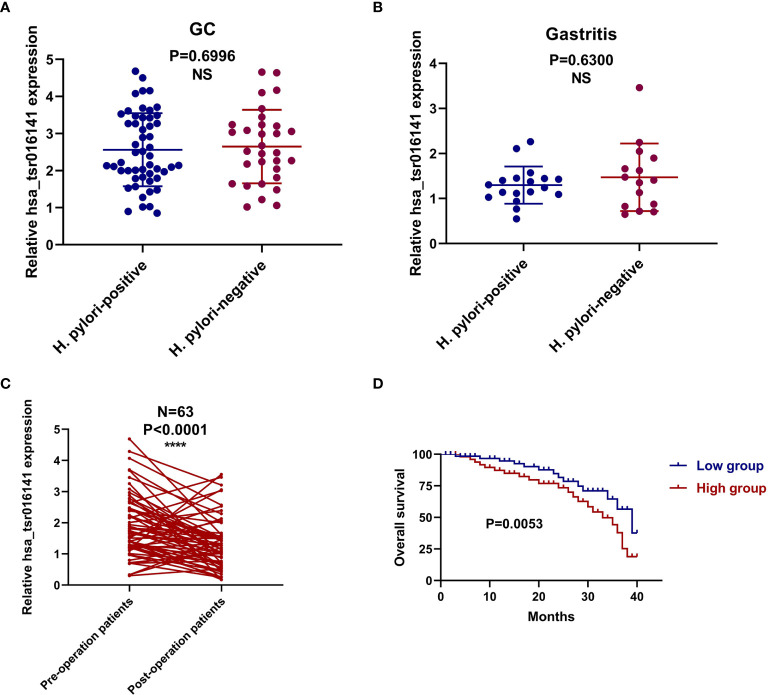
The corresponding relationship with Helicobacter pylori infection and prognostic value of hsa_tsr016141 in GC. **(A)** The expression level of hsa_tsr016141 between the Helicobacter pylori-positive and Helicobacter pylori-negative groups in GC patients. **(B)** The expression level of hsa_tsr016141 between the Helicobacter pylori-positive and Helicobacter pylori-negative groups in gastritis patients. **(C)** The expression levels of the serum hsa_tsr016141 in the 63 GC patients before and after the operation. **(D)** Survival curve verifies the prognostic value of hsa_tsr016141. ****P < 0.0001, NS P > 0.05.

In order to investigate the clinical application value of serum hsa_tsr016141, we collected the clinicopathological data of 130 patients with GC. We divided them into two groups according to the median: higher expression group (expression>1.725, n=65) and lower expression group (expression<1.725, n=65). As shown in **Table 6**, the expression level of hsa_tsr016141 was positively correlated with tumor differentiation grade, T stage, lymph node status, and TNM stage, but not with sex, age, tumor size, and nerve/vascular invasion. The occurrence of GC is caused by many factors, including helicobacter pylori infection, precancerous lesions, diet, or genetic factors. Among them, helicobacter pylori chronic infection is a major cause of stomach cancer, accounting for about 89% of the cases of distal GC in the world (25–27). In order to explore whether the expression level of hsa_tsr016141 is related to Helicobacter pylori infection, we conducted induction analysis in 130 GC patients and found that 83 of 130 GC patients were tested for Helicobacter pylori, of which 52 were positive, with a positive rate of 64.6%. Then, we statistically analyzed the expression level of hsa_tsr016141 in the Helicobacter pylori-positive group and Helicobacter pylori-negative group and found no significant difference in hsa_tsr016141 expression level between the Helicobacter pylori-positive group and Helicobacter pylori-negative group. Similarly, we conducted the same analysis on 50 patients with gastritis. Among the 50 patients with gastritis, 33 cases were tested for Helicobacter pylori, 18 were positive, the positive rate was 54.5%. There was no significant difference in the expression level of hsa_tsr016141 between the Helicobacter pylori-positive and Helicobacter pylori-negative groups in patients with gastritis which indicated that there was no significant correlation between Helicobacter pylori-positive group and hsa_tsr016141 expression level **(** **Figures 6A, B** **)**.

**Table 6:** Clinicopathological analysis of hsa_tsr016141.

Parameter		No. of patients	hsa_tsr016141(high)	hsa_tsr016141(low)	P-value
**Sex**	male	94	46	48	0.7907
****	female	36	19	17	
**Age(year)**	<60	40	19	21	0.2111
****	≥60	90	46	44	
**Tumor size**	<5	96	43	53	0.0705
****	≥5	34	22	12	
**Differentiation grade**	Well-moderate	63	24	39	0.0006***
****	Poor-undifferentiation	67	41	26	
**T stage**	T1-T2	72	26	46	0.0004***
****	T3-T4	58	39	19	
**Lymph node status**	Positive	78	45	33	0.0047**
****	Negative	52	20	32	
**TNM stage**	I-II	74	29	45	0.0001***
****	III-IV	56	36	20	
**Nerve/vascular invasion**	Positive	77	42	35	0.0815
	Negative	53	23	30	

*P < 0.05, **P < 0.01, ***P < 0.001, ****P < 0.0001.

### The Role of Serum hsa_tsr016141 in Dynamic Monitoring of Tumor in Patients With GC

In order to verify the dynamic relationship between serum hsa_tsr016141 expression level and tumor progression, we compared the difference of hsa_tsr016141 expression level before and after surgery in 63 patients with GC. It was found that the serum hsa_tsr016141 expression decreased significantly in the same patient after GC surgery **(** **Figure 6C** **)**. In addition, the survival curve showed that the survival rate of the low expression group was higher than that of the high expression group, indicating that hsa_tsr016141 can effectively track the postoperative condition of GC and dynamically monitor the patients with GC **(** **Figure 6D** **)**.

## Discussion

GC is one of the most serve malignant tumors in the world, with the highest mortality rate. However, nowadays, tumor markers that can be applied to GC have low sensitivity and specificity, and most patients have reached an advanced stage when GC is found (28). Therefore, it is urgent to find biomarkers with high sensitivity and specificity suitable for the early screening of GC.

tsRNAs are a new member of the family of small non-coding RNAs. Their stable structure and high abundance in body fluids show their potential to be used in liquid biopsy and become a new generation of tumor biomarkers. At present, most of the studies on tsRNAs have described the effects of tsRNAs on tumor cell proliferation, invasion, and migration, as well as their internal pathways and mechanisms. However, there are still few studies on whether tsRNAs can be applied as biomarkers (17, 29, 30). Therefore, in this study, we explored the potential of hsa_tsr016141 as a tumor marker for GC.

We screened out three 5’tRF and 3’tRF with the condition of biggest log_2_ fold change and P<0.05 through the OncotRF database. By comparing the expression of 5’tRF and 3’tRF in 20 pairs of GC tissues and their matching adjacent non-tumor tissues, we selected the most differentially expressed 5’tRF and named it hsa_tsr016141 according to the naming rule of tsRBase. Then we studied its molecular characteristics, confirmed its chromosome location, and verified the PCR amplification products by AGE and Sanger sequencing. Then, through experiments, we found that its stability and specificity were good, and it was mainly located in the nucleus. Subsequently, we found that it was secreted continuously from tumor cells over time, showing its potential as a biomarker. Then, we verified that the detection method of hsa_tsr016141 could be applied to clinical analysis. By analyzing a large sample of hsa_tsr016141, we found that the expression level of hsa_tsr016141 was gradient change in GC patients, gastritis patients, and healthy donors. By classifying the expression levels of these 130 GC patients by N stage and TNM stage, we found that with the increasing of lymph node metastasis and the increasing tumor grade, the expression level of hsa_tsr016141 also increased. After ROC analysis, it was found that hsa_tsr016141 could significantly distinguish GC patients from healthy donors as well as GC patients from gastritis patients. The sensitivity of hsa_tsr016141, CEA, and CA199 in the joint diagnosis of GC is 90%, while the sensitivity of hsa_tsr016141 and CA724 in the joint diagnosis of GC is 82%. After the analysis of clinicopathological data, it was found that the expression level of hsa_tsr016141 was correlated with differentiation grade, T stage, lymph node status, and TNM stage. By comparing the expression level of hsa_tsr016141 in Helicobacter pylori-positive and Helicobacter pylori-negative GC patients and gastritis patients, we found that there was no significant correlation between Helicobacter pylori infection and the expression level of hsa_tsr016141. In addition, through the analysis of the survival curve and the expression level of hsa_tsr016141 in patients with GC patients after operation, we found that hsa_tsr016141 could dynamically monitor the patients with GC after the operation.

Several previous studies have highlighted the potential use of tRFs as biomarkers for diagnosing different types of cancer (16, 31, 32). For example, Mo found that the serum expression level of 5’-tiRNA ^Val^ was significantly decreased in 60 breast cancer patients compared with normal controls, suggesting that it may inhibit the progression of breast cancer (16). Shen detected the expression level of tRF-33-P4R8YP9LON4VDP in 89 gastric cancer patients and 98 healthy plasma samples. The expression level of tRF-33-P4R8YP9LON4VDP in plasma of gastric cancer patients was significantly lower than that of normal people (32). They have verified the potential of tRFs as tumor biomarkers, but they all have limitations and lack of validation of molecular properties and detection methods. In our research, we conducted a comprehensive analysis of the potential of hsa_tsr016141 as a biomarker for GC, the elevated serum hsa_tsr016141 has good stability and specificity, which makes it have the potential to be used as a good biomarker for GC. Meanwhile, the elevated serum hsa_tsr016141 can effectively track the postoperative situation of GC, dynamically monitor GC patients. Besides, the increased expression level of hsa_tsr016141 is positively correlated with lymph node metastasis and tumor grade, which has high diagnostic efficacy for GC. Its detection method also has good clinical application value. In conclusion, we found that hsa_tsr016141 could be used for the diagnosis and postoperative monitoring of GC patients. In future studies, larger samples are needed to verify the value of the clinical application.

tsRNAs are divided into tRFs and tiRNAs, and tRFs were divided into 5’tRF and 3’tRF according to the different digesting positions. Previous research has shown that 5’tRF is mainly present in the nucleus (33), while 3’tRF is mainly present in the cytoplasm (34). In this study, we found that hsa_tsr016141 belongs to 5’tRF and is mainly located in the nucleus as previous studies had concluded. Some studies have found that tsRNAs can inhibit the translation process (35, 36). For instance, 3’tRF from tRNA^Leu-CAG^ of non-small cell lung cancer (NSCLC) cells has a similar effect to miRNA and can attenuate protein translation (35). Another study found that 5‘tiRNA^Ala^ and 5’ tiRNA^Cys^ could inhibit translation by forming intermolecular RNA G quadruplexes (RG4) to replace the translation initiation complex eIF4G/eIF4E which located on mRNA cap (36). Simultaneously, some tsRNAs can influence the occurrence and progression of diseases through the protein sponge mechanism (37, 38). For example, a series of tRFs generated by Glu/Asp/Gly/Tyr tRNAs in the low metastatic breast cancer cell line MDA under hypoxia conditions competitively bind with oncogene YBX1 protein, which reduces the stability of oncogene transcripts and thus inhibits cancer metastasis (37). When it comes to 5’tRF, some researchers believe that both 5’tRF and 3’tRF are involved in RNA silencing, while others believe that 5’tRF can inhibit the protein translation process and is considered a novel gene regulation mechanism (39, 40). Therefore, we predict that the regulation of hsa_tsr016141 on GC might affect protein translation or RNA silencing by binding to downstream mRNA. To our knowledge, this is the first report to elucidate that hsa_tsr016141 could be used in the diagnosis and postoperative monitoring of GC, and there are still no reports on the functional role of hsa_tsr016141 in other types of cancer also. However, the specific mechanism of hsa_tsr016141 is still unclear, which needs to be further studied and verified in the future.

## Data Availability Statement

The original contributions presented in the study are included in the article/supplementary material. Further inquiries can be directed to the corresponding authors.

## Ethics Statement

The studies involving human participants were reviewed and approved by The ethics committee of the local hospital (ethical review report number: 2018-L055). The patients/participants provided their written informed consent to participate in this study.

## Author Contributions

Material preparation, data collection and analysis were performed by XG and SM. The first draft of the manuscript was written by XG, resources and guidance for the paper were provided by BL and SJ, and all authors commented on previous versions of the manuscript. All authors contributed to the article and approved the submitted version.

## Funding

This project was supported by the National Natural Science Foundation of China (No. 81871720, 82072363).

## Conflict of Interest

The authors declare that the research was conducted in the absence of any commercial or financial relationships that could be construed as a potential conflict of interest.

## Abbreviations

GC, Gastric cancer; BC, Breast cancer; CRC, Colorectal cancer; LC, Lung cancer; TC, Thyroid cancer; tsRNAs, tRNA-derived small RNAs; tRFs, tRNA-derived fragments; tiRNAs, tRNA halves; AGE, agarose gel electrophoresis; CV, coefficient of variation; CEA, carcinoembryonic antigen; CA199, Carbohydrate antigen199; CI, confidence interval; CA724, Carbohydrate antigen724; NSCLC, non-small cell lung cancer.

## References

[1] Thrift A El-Serag H. Burden of Gastric Cancer. Clin gastroenterology hepatology Off Clin Pract J Am Gastroenterological Assoc (2020) 18(3):534–42. 10.1016/j.cgh.2019.07.045 PMC8859863 31362118

[2] Karimi P Islami F Anandasabapathy S Freedman N Kamangar F. Gastric Cancer: Descriptive Epidemiology, Risk Factors, Screening, and Prevention. Cancer epidemiology Biomarkers Prev Publ Am Assoc Cancer Research cosponsored by Am Soc Prev Oncol (2014) 23(5):700–13. 10.1158/1055-9965.Epi-13-1057 PMC4019373 24618998

[3] Ferlay J Colombet M Soerjomataram I Mathers C Parkin D Piñeros M. Estimating the Global Cancer Incidence and Mortality in 2018: GLOBOCAN Sources and Methods. Int J Cancer (2019) 144(8):1941–53. 10.1002/ijc.31937 30350310

[4] Ma S Kong S Wang F Ju S. Circrnas: Biogenesis, Functions, and Role in Drug-Resistant Tumours. Mol Cancer (2020) 19(1):119. 10.1186/s12943-020-01231-4 32758239 PMC7409473

[5] Sun L Jiang R Li J Wang B Ma C Lv Y. Micorna-425-5p is a Potential Prognostic Biomarker for Cervical Cancer. Ann Clin Biochem (2017) 54(1):127–33. 10.1177/0004563216649377 27166306

[6] Mu L Wang Y Su H Lin Y Sui W Yu X. HIF1A-AS2 Promotes the Proliferation and Metastasis of Gastric Cancer Cells Through Mir-429/PD-L1 Axis. Digestive Dis Sci (2021) S1525–0016(21):00065–4. 10.1007/s10620-020-06819-w 33555514

[7] Zhu J Luo Y Zhao Y Kong Y Zheng H Li Y. Circehbp1 Promotes Lymphangiogenesis and Lymphatic Metastasis of Bladder Cancer Via Mir-130a-3p/Tgfβr1/VEGF-D Signaling. Mol Ther J Am Soc Gene Ther (2021) S1525-0016(21):00065–4. 10.1016/j.ymthe.2021.01.031 PMC8116613 33545359

[8] Borek E Baliga B Gehrke C Kuo C Belman S Troll W. High Turnover Rate of Transfer RNA in Tumor Tissue. Cancer Res (1977) 37(9):3362–6. 884680

[9] Speer J Gehrke C Kuo K Waalkes T Borek E. Trna Breakdown Products as Markers for Cancer. Cancer (1979) 44(6):2120–3. 10.1002/1097-0142(197912)44:6<2120::aid-cncr2820440623>3.0.co;2-6 509391

[10] Lee R Feinbaum R Ambros V. The C. Elegans Heterochronic Gene Lin-4 Encodes Small Rnas With Antisense Complementarity to Lin-14. Cell (1993) 75(5):843–54. 10.1016/0092-8674(93)90529-y 8252621

[11] Wightman B Ha I Ruvkun G. Posttranscriptional Regulation of the Heterochronic Gene Lin-14 by Lin-4 Mediates Temporal Pattern Formation in C. Elegans. Cell (1993) 75(5):855–62. 10.1016/0092-8674(93)90530-4 8252622

[12] Zhu P Yu J Zhou P. Role of Trna-Derived Fragments in Cancer: Novel Diagnostic and Therapeutic Targets Trfs in Cancer. Am J Cancer Res (2020) 10(2):393–402. 32195016 PMC7061753

[13] Shen Y Yu X Zhu L Li T Yan Z Guo J. Transfer RNA-Derived Fragments and Trna Halves: Biogenesis, Biological Functions and Their Roles in Diseases. J Mol Med (Berlin Germany) (2018) 96(11):1167–76. 10.1007/s00109-018-1693-y 30232504

[14] Zhu L Ge J Li T Shen Y Guo J. Trna-Derived Fragments and Trna Halves: The New Players in Cancers. Cancer Lett (2019) 452:31–7. 10.1016/j.canlet.2019.03.012 30905816

[15] Xie Y Yao L Yu X Ruan Y Li Z Guo J. Action Mechanisms and Research Methods of Trna-Derived Small Rnas. Signal transduction targeted Ther (2020) 5(1):109. 10.1038/s41392-020-00217-4 PMC7326991 32606362

[16] Mo D Jiang P Yang Y Mao X Tan X Tang X. A Trna Fragment, 5’-Tirna, Suppresses the Wnt/Beta -Catenin Signaling Pathway by Targeting FZD3 in Breast Cancer. Cancer Lett (2019) 457:60–73. 10.1016/j.canlet.2019.05.007 31078732

[17] Zhang F Shi J Wu Z Gao P Zhang W Qu B. A 3’-Trna-Derived Fragment Enhances Cell Proliferation, Migration and Invasion in Gastric Cancer by Targeting FBXO47. Arch Biochem biophysics (2020) 690:108467. 10.1016/j.abb.2020.108467 32592804

[18] Tong L Zhang W Qu B Zhang F Wu Z Shi J. The Trna-Derived Fragment-3017A Promotes Metastasis by Inhibiting NELL2 in Human Gastric Cancer. Front Oncol (2020) 10:570916. 10.3389/fonc.2020.570916 33665159 PMC7921707

[19] Zong T Yang Y Zhao H Li L Liu M Fu X. Tsrnas: Novel Small Molecules From Cell Function and Regulatory Mechanism to Therapeutic Targets. Cell proliferation (2021) 54(3):e12977. 10.1111/cpr.12977 33507586 PMC7941233

[20] Couvillion M Sachidanandam R Collins K. A Growth-Essential Tetrahymena Piwi Protein Carries Trna Fragment Cargo. Genes Dev (2010) 24(24):2742–7. 10.1101/gad.1996210 PMC3003190 21106669

[21] Zhu L Liu X Pu W Peng Y. Trna-Derived Small Non-Coding Rnas in Human Disease. Cancer Lett (2018) 419:1–7. 10.1016/j.canlet.2018.01.015 29337107

[22] Kim D Oh S Oh C Choi M Noh J Sohn T. The Relationships Between Perioperative CEA, CA 19-9, and CA 72-4 and Recurrence in Gastric Cancer Patients After Curative Radical Gastrectomy. J Surg Oncol (2011) 104(6):585–91. 10.1002/jso.21919 21695697

[23] Guadagni F Roselli M Cosimelli M Ferroni P Spila A Casaldi V. Correlation Between Positive CA 72-4 Serum Levels and Lymph Node Involvement in Patients With Gastric Carcinoma. Anticancer Res (1993) 13:2409–13. 8135475

[24] Aloe S D’Alessandro R Spila A Ferroni P Basili S Palmirotta R. Prognostic Value of Serum and Tumor Tissue CA 72-4 Content in Gastric Cancer. Int J Biol Markers (2003) 18(1):21–7. 10.5301/jbm.2008.1151 12699059

[25] González C Megraud F Buissonniere A Lujan Barroso L Agudo A Duell E. Helicobacter Pylori Infection Assessed by ELISA and by Immunoblot and Noncardia Gastric Cancer Risk in a Prospective Study: The Eurgast-EPIC Project. Ann Oncol Off J Eur Soc Med Oncol (2012) 23(5):1320–4. 10.1093/annonc/mdr384 21917738

[26] Lochhead P El-Omar E. Helicobacter Pylori Infection and Gastric Cancer. Best Pract Res Clin gastroenterology (2007) 21(2):281–97. 10.1016/j.bpg.2007.02.002 17382277

[27] Plummer M Franceschi S Vignat J Forman D de Martel C. Global Burden of Gastric Cancer Attributable to Helicobacter Pylori. Int J Cancer (2015) 136(2):487–90. 10.1002/ijc.28999 24889903

[28] Hamashima C. Current Issues and Future Perspectives of Gastric Cancer Screening. World J gastroenterology (2014) 20(38):13767–74. 10.3748/wjg.v20.i38.13767 PMC4194560 25320514

[29] Zhang M Li F Wang J He W Li Y Li H. Trna-Derived Fragment Trf-03357 Promotes Cell Proliferation, Migration and Invasion in High-Grade Serous Ovarian Cancer. OncoTargets Ther (2019) 12:6371–83. 10.2147/ott.S206861 PMC6702494 31496739

[30] Zhu J Cheng M Zhao X. A Trna-Derived Fragment (Trf-3001b) Aggravates the Development of Nonalcoholic Fatty Liver Disease by Inhibiting Autophagy. Life Sci (2020) 257:118125. 10.1016/j.lfs.2020.118125 32702444

[31] Zhu L Li T Shen Y Yu X Xiao B Guo J. Using Trna Halves as Novel Biomarkers for the Diagnosis of Gastric Cancer. Cancer Biomarkers section A Dis Markers (2019) 25(2):169–76. 10.3233/cbm-182184 PMC13082404 31104009

[32] Shen Y Yu X Ruan Y Li Z Xie Y Yan Z. Global Profile of Trna-Derived Small Rnas in Gastric Cancer Patient Plasma and Identification of Trf-33-P4R8YP9LON4VDP as a New Tumor Suppressor. Int J Med Sci (2021) 18(7):1570–9. 10.7150/ijms.53220 PMC7976566 33746573

[33] Kumar P Kuscu C Dutta A. Biogenesis and Function of Transfer RNA-Related Fragments (Trfs). Trends Biochem Sci (2016) 41(8):679–89. 10.1016/j.tibs.2016.05.004 PMC5173347 27263052

[34] Kumar P Anaya J Mudunuri S Dutta A. Meta-Analysis of Trna Derived RNA Fragments Reveals That They are Evolutionarily Conserved and Associate With AGO Proteins to Recognize Specific RNA Targets. BMC Biol (2014) 12:78. 10.1186/s12915-014-0078-0 25270025 PMC4203973

[35] Shao Y Sun Q Liu X Wang P Wu R Ma Z. Trf-Leu-CAG Promotes Cell Proliferation and Cell Cycle in Non-Small Cell Lung Cancer. Chem Biol Drug design (2017) 90(5):730–8. 10.1111/cbdd.12994 PMC5697697 28378898

[36] Kuscu C Kumar P Kiran M Su Z Malik A Dutta A. Trna Fragments (Trfs) Guide Ago to Regulate Gene Expression Post-Transcriptionally in a Dicer-Independent Manner. RNA (New York NY) (2018) 24(8):1093–105. 10.1261/rna.066126.118 PMC6049499 29844106

[37] Goodarzi H Liu X Nguyen H Zhang S Fish L Tavazoie S. Endogenous Trna-Derived Fragments Suppress Breast Cancer Progression Via YBX1 Displacement. Cell (2015) 161(4):790–802. 10.1016/j.cell.2015.02.053 25957686 PMC4457382

[38] Krishna S Yim D Lakshmanan V Tirumalai V Koh J Park J. Dynamic Expression of Trna-Derived Small Rnas Define Cellular States. EMBO Rep (2019) 20(7):e47789. 10.15252/embr.201947789 31267708 PMC6607006

[39] Burroughs A Ando Y de Hoon M Tomaru Y Suzuki H Hayashizaki Y. Deep-Sequencing of Human Argonaute-Associated Small Rnas Provides Insight Into Mirna Sorting and Reveals Argonaute Association With RNA Fragments of Diverse Origin. RNA Biol (2011) 8(1):158–77. 10.4161/rna.8.1.14300 PMC3127082 21282978

[40] Sobala A Hutvagner G. Small Rnas Derived From the 5’ End of Trna Can Inhibit Protein Translation in Human Cells. RNA Biol (2013) 10(4):553–63. 10.4161/rna.24285 PMC3710361 23563448

